# The effects of aqueous extract of *Labisia Pumila (Blume) Fern.-Vill. Var. Alata* on wound contraction, hydroxyproline content and histological assessments in superficial partial thickness of second-degree burn model

**DOI:** 10.3389/fphar.2022.968664

**Published:** 2022-10-12

**Authors:** Nurul ‘Izzah Ibrahim, Isa Naina Mohamed, Norazlina Mohamed, Elvy Suhana Mohd Ramli, Ahmad Nazrun Shuid

**Affiliations:** ^1^ Department of Pharmacology, Faculty of Medicine, Universiti Kebangsaan Malaysia Medical Centre, Kuala Lumpur, Malaysia; ^2^ Department of Anatomy, Faculty of Medicine, Universiti Kebangsaan Malaysia Medical Centre, , Kuala Lumpur, Malaysia; ^3^ Department of Pharmacology, Faculty of Medicine, Universiti Teknologi MARA, Sungai Buloh Campus, Jalan Hospital, Sungai Buloh, Selangor, Malaysia

**Keywords:** labisia pumila, antioxidant, burn wound healing, hydroxyproline, histomorphology

## Abstract

**Background:** Burns are considered a critical care problem in emergency medicine, resulting in physical, psychological, and chronic disabilities. Silver sulfadiazine is the gold standard in topical burn treatment but was associated with toxicity to keratinocytes and fibroblasts, which may delay wound healing. In discovering potential alternative treatments for burn wound healing, this study was performed to determine the effect of *Labisia Pumila (Blume) Fern.-Vill. Var. Alata* (LPVa) extract on thermal-burn wounds in rats.

**Methods:** A total of 50 *Sprague-Dawley* male rats were categorized into five groups. There were three control groups; normal control (left untreated), negative control (given ointment base) and positive control (given silver sulfadiazine). Meanwhile, the two intervention groups were given with 2% LPVa leaf and root extracts, respectively. Burn wounds were inflicted on the loin region of the rat by applying a heated steel rod at 80°C for 10 s. On days 3, 7, 14, and 21, wounds were measured macroscopically using a digital calliper and one animals of each group were sacrificed, and the wounded skin were excised for histomorphological assessments. The wounds were excised for hydroxyproline content on Day 14 of treatment.

**Result:** For wound contraction percentage, both the leaf and root extracts of LPVa showed a significant reduction in burn wound size on Day 7 onwards, when compared to other groups. For hydroxyproline content, only the leaf extract of LPVa produced significantly higher content compared to both negative and normal control groups. In terms of histological examination, the leaf extract group demonstrated a superior healing effect than the root extract group.

**Conclusion:** Both leaf and root extracts of LPVa could promote wound healing in the thermal-burn wound rat model, with leaf extract being superior to root extract.

## Introduction

Burns are a critical care problem requiring specialised care focusing on stabilising the patient, preventing infection, and optimizing functional recovery (Rowan et al., 2015). Burns can be defined as tissue lesions that occur as a result of exposure to thermal origin such as flames, hot surface and liquids, extreme cold, chemicals, radiation or friction (Tavares Pereira et al., 2012). Burns are categorised according to the severity of lesion into first, second and third-degree of burns. For the first-degree burn, it is restricted to the epidermal layer that results into redness and require simple first-aid procedures with over-the-counter pain relievers. Second-degree burn or also known as partial-thickness burns are subdivided into two categories: superficial and deep. As for the superficial partial thickness, the burn may reach the epidermis and superficial dermis, causing hypersensitivity and pain. Deep partial-thickness burn occurs when it reaches the deepest layer of the dermis, resulting in reduced sensitivity with red and/or white colouration of the tissue. Finally, the third-degree burn or also known as full-thickness burn involves the subcutaneous layer, without sensitivity and white in colour (Lanham et al., 2020).

Burn wound healing is a complex process that can be divided into three overlapping phases: inflammatory, proliferative and remodelling phases. During the inflammatory phase, neutrophils and monocytes arrive at the injury site *via* localized vasodilation and fluid extravasation (Tiwari, 2012). The inflammation prevents wound infection, degrades necrotic tissue and activate signals required for wound repair (Reinke and Sorg, 2012). The next overlapping phase is known as the proliferative phase, which is characterized by activation of keratinocyte and fibroblast by cytokines and growth factors (Werner et al., 2007). During this phase, keratinocytes, the dominant cell types in epidermis layer will migrate over the wound to aid in closure and restoration of a vascular network that results into maturation of epidermis and scar. The communication between stromal, endothelial and immune cells may define the healing course of the wound such as closure and revascularization (Pastar et al., 2014).

The healing of burn-wound lesions involves tissue inflammation, oedema, and hypertrophic scars (Rowan et al., 2015). Hence, the type of topical agent or coverage for treating burns should be selected based on assessment of lesion characteristics and evidence reported by specific literatures. Topical agents used for treating burn wounds should have properties such as antimicrobial activity, good compliance and cheap. They should also be less toxic, produce less hypersensitivity reactions and able to shorten the healing time (Tavares Pereira et al., 2012).

Silver sulfadiazine is the gold standard for topical burn treatment. It improves the survival of patients with major burns and minimizes the incidence of burn wound sepsis, a leading cause of mortality and morbidity in burn patients (Church et al., 2006). It is composed of sodium sulfadiazine and silver nitrate, whereby the silver ion attaches to the nucleic acid of the microorganisms, releasing the sulfadiazine. The sulfadiazine produced from the reaction will then disrupt the metabolism of the microbe (Lansdown, 2002b). However, the silver constituent in the silver sulfadiazine has been demonstrated to interact with structural proteins and preferentially bind to DNA nucleic acid bases to inhibit replication of the cells in the skin (Lansdown, 2002a; 2002b). Due to this reaction, silver is toxic to keratinocytes and fibroblasts, which may cause delay in burn wound healing if applied continuously to the healing tissue areas (Nešporová et al., 2020). Thus, an alternative treatment that could promote burn wound healing effectively but less toxic needs to be developed. The treatment of burn wounds has evolved over several decades through clinical and preclinical research. Significant advancements have been made in treatment of burn wound, including the testing of unique pharmacological interventions such as herbal plant extracts.

*Labisia pumila (Blume) Fern.-Vill. (Primulaceae)* (LP) or locally known as ‘Kacip Fatimah’ is one of the herbal plants in the *Myrsinaceae* family, characterized by lanceolate leaves with a creeping stem and long roots (Jamal et al., 2003). The plant is commonly found in the South East Asian region, including Malaysia, Indonesia, Thailand, and China (Stone, 1989). LP is a jungle species and difficult to cultivate *ex situ*, which requires adaptation to the agricultural environment before it can survive and grow optimally. Therefore, LP does not associate with seasonal collection of the plants. In Malaysia, for instance, the plant raw materials come directly from the jungle or are imported from neighbouring countries. (Rosnani et al., 2019). LP is a well-known and widely used herbal remedy by women for general well-being and specifically to facilitate and expedite recovery after childbirth. This includes expediting healing of childbirth wounds. Its pharmacological effects on women’s health may be related to its phytoestrogen properties, having similar chemical structure to estrogen (Jamal et al., 2003). Phytoestrogens are regarded as the naturally occurring selective estrogen receptor modulators (SERMs) and possess potential effect in providing a natural estrogen replacement especially to postmenopausal women. Phytoestrogens demonstrated protection against oxidative stress, an imbalanced condition between ROS and antioxidant defence mechanism (Liu et al., 2020). There are three known varieties of LP which are var. *pumila,* var. *alata*, and var. *lanceolata* (Karimi et al., 2013). The var. *alata* and var. *pumila* variants were more commonly used as medicinal plants than var. *lanceolata* (Abdul Kadir et al., 2012)*.* Several studies have demonstrated that LP extracts possess medicinal properties such as antifungal, anti-inflammatory, cytotoxicity (Karimi et al., 2013), anticancer, antioxidant and anti-osteoporosis (Nadia et al., 2012). However, there are limited studies on LP effects on skin tissue. In a previous study by Choi et al. (2010), LP extract demonstrated protection of skin against photoaging induced by ultraviolet irradiation (Choi et al., 2010). Meanwhile, a study by Ahmad et al. (2018) reported that LP extract promoted minor wound healing in ovariectomized rat model (Ahmad et al., 2018). However, the effects of LP on burn wound healing have not been studied yet. To the best of our knowledge, this is the first study aimed to determine the potential of LP var alata in promoting burn wound healing.

## Materials and methods

### Preparation of *Labisia Pumila* extracts and combination with ointment

Standardised methods were used to obtain aqueous extracts of leaf and roots of *Labisia pumila var. alata* (Jamal et al., 2003). Briefly, the extraction was performed with water by successive maceration at room temperature for a week, followed by filtration. The filtration process was repeated several times. The filtrate obtained after filtration were concentrated by evaporation using a rotary evaporator (Buchi Rotavapor R-100, Switzerland) at temperatures of 35°C until dryness to maximize the proportion of desired bioactive fractions contained in each of the extract. The filtrate was then freeze-dried to obtain the powdered form. The process of extraction, filtration and concentration were repeated several times until maximum yield of aqueous extracts has been reached.

The LP leaf and root aqueous extracts were combined with cetomacrogol emulsifying ointment (Hovid Berhad, Malaysia), which is a type of paraffin used as a vehicle in these topically-applied preparations to the rats. This emulsifying ointment was chosen as a vehicle as it is chemically inert and inactive to the skin (Ahmad et al., 2021). A 2.0% dose concentration was chosen for both the leaf and root extracts as previously established by Ahmad et al. (2018) using LP extracts on minor wound healing (Ahmad et al., 2018). The extracts were crushed using a pestle and mortar to obtain fine powder which have faster absorption rates and better uniformity when mixed with emulsifying ointment. In brief, the emulsifying ointment and extract powder were put together onto a clean glass plate and mixed thoroughly with a spatula to ensure uniformity. The extract appeared dissolved during the mixing process, which confirmed its compatibility with the ointment.

### Animals

Fifty male Sprague-Dawley rats aged 4–5 months and weighing 250 ± 50 g were obtained from the Universiti Kebangsaan Malaysia Laboratory Animal Research Unit. All rats were housed in individually ventilated cages at temperature-controlled (25 ± 1°C) environment under natural day/night cycle. They were fed with standard laboratory food pellets and given water *ad libitum*. All animal experiments were approved by Universiti Kebangsaan Malaysia Animal Ethical Committee (FAR/PP/2018/NAZRUN/25-JULY/935-AUG-2018-MAR-2019).

### Burn wound model and treatments

**FIGURE 1:**
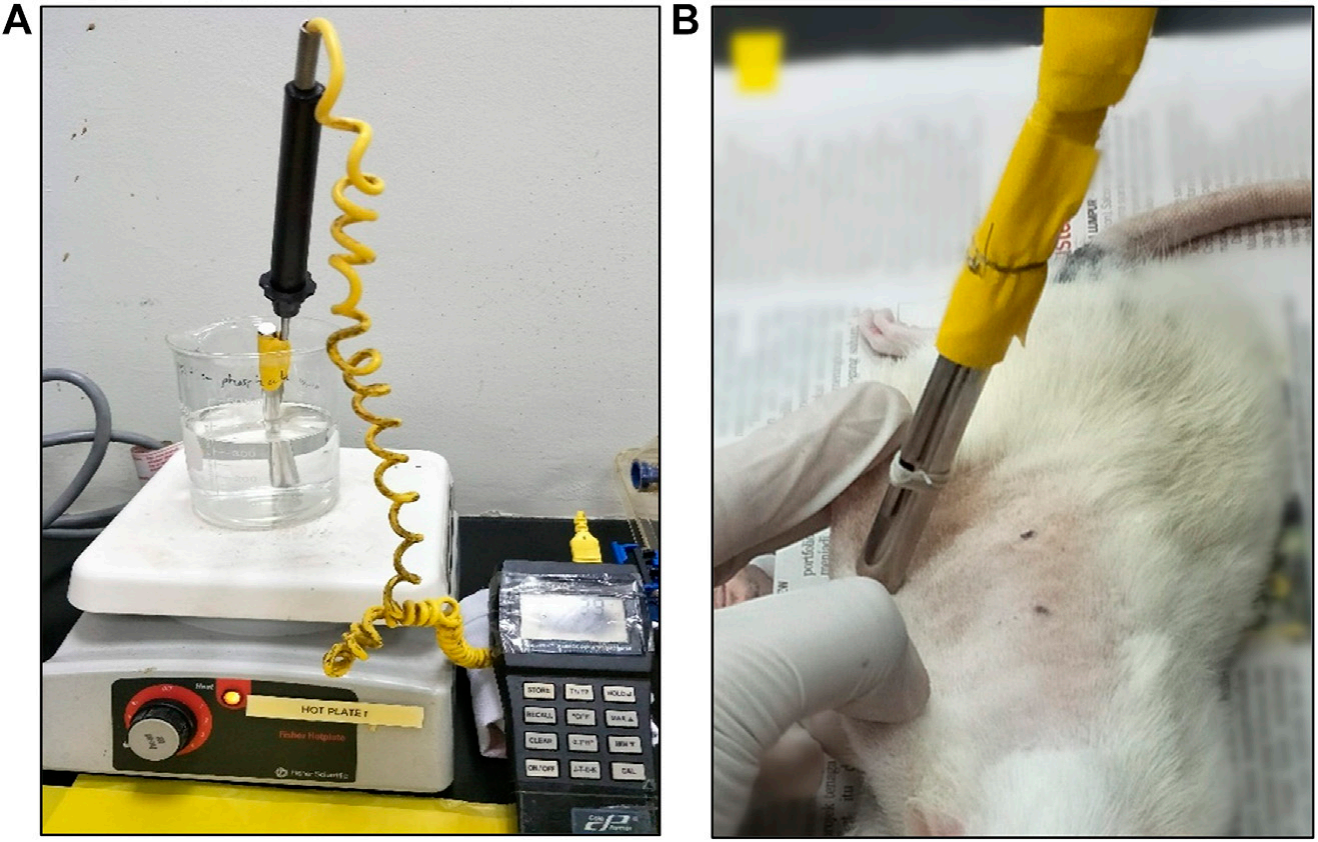
**(A)** Configuration of thermocouple thermometer and boiling water. **(B)** Experimental burn wound infliction performed on a rat.

The rats were acclimatized to laboratory conditions for 1 week prior to the experiment. Initially, the rats were anesthetized with intraperitoneal injection of Ketamine and Xylazil at 1:1 ratio before inducing burn wound. The dorsal regions of the rats were shaven with an electric shaver and sterilized with 70% alcohol. Prior to burn-wound infliction, the rats were injected intramuscularly with tramadol (12.5 mg/kg body weight) for pain control. Burn infliction techniques were performed accordingly to the methods by Cai et al. (2014) with slight modification (Cai et al., 2014). Burn wounds were created at dorsum of the rats using a 100 g cylindrical stainless-steel rod (1 cm diameter), which was heated to 80°C in boiling water. Temperature was monitored using a thermocouple thermometer (Figure 1A). The area for burn infliction was limited to the loin region of the rat. Four points were marked for burn wound sites at the loin with 2 cm apart side to side and 4 cm up and down. Then, the skin was pulled upwards, away from the underlying viscera, creating a flat surface (Figure 1B). The rod was left rested on its own weight for 10 s at the four different sites on each rat. Treatments were given topically to the rats, which were categorized into five groups, each containing 10 rats. As for the control groups, the wounds were left untreated for normal control (NM) group, treated with emulsifying ointment for negative control (NG) group and treated with silver sulfadiazine for positive control (PS) group. For the two intervention groups, the wounds were treated with LPVa leaf (LF) and root (RT) extracts, respectively. Treatments were performed for 3 weeks on daily basis.

### Macroscopic evaluation of burn wounds

Following the burn infliction, macroscopic changes of burn wounds were evaluated at day 0 post burn and subsequently at days 3, 7, 14, and 21 post burn. Photographs were taken with digital camera (SONY DSC-RX100M2, 15x) until complete closure of wound. The images were scored blindly by anatomical expert using a macroscopic scoring tool for burns as described by Schlager et al. (2000)(Schlager et al., 2000) (Table 1).

**TABLE 1:** Macroscopic scoring system used for burns. Source: Schlager et al. (2000).

Variable	Scoring		
	0	1	2
Redness	None	Slightly red	Completely red
Edema	None	Minimal edema	Clearly distinctive

### Measurement of wound area

**FIGURE 2:**
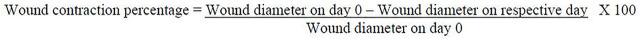
Formula for calculating reduction in percentage of the original wound size.

Wounds were observed and measured at day 0, 3, 7, 14, and 21 by using a digital caliper (General Ultratech, New York, NY). Woud contraction will be expressed as reduction in percentage of the original wound size (Figure 2).

### Determination of the hydroxyproline content

On the 14th day, one rat from each group was euthanized using ketamine and xylazil (overdose) to excise the wound tissue for determination of hydroxyproline content. The protein content of the wound tissue was estimated using the techniques described by Neuman and Logan (1950) (Neuman and Logan, 1950). Wound tissue were excised and stored at −70°C until ready for processing. For the procedure, 80–100 mg of tissues were weighed and minced to small pieces and were put in test tubes. Tissues were hydrolysed by adding 6 mol/L HCl to each test tubes and placed in boiling water bath for 5 h. Then, the pH of the hydrolysate was adjusted between 6.0 and 6.8 by testing with pH indicator paper. Distilled water was added into each test tubes to a final volume of 10 ml and they were mixed thoroughly. Approximately 3–4 ml of diluted test solution was taken and added with 20–30 mg of activated carbon. The solution was mixed thoroughly and centrifuged at 3,500 rpm for 10 min. For detection, 1 ml of the test solution was taken and prepared according to the manual given in the test kit. Blank and standard tubes were also prepared accordingly. After mixing the solution thoroughly, the mixture was incubated at 60°C in water bath for 15 min and centrifuged at 3,500 rpm for 10 min. Supernatants were taken and measured using ELISA plate reader at 550 nm wavelength.

### Histomorphological analysis

Three types of staining methods were used for histomorphological analysis; 1) Hematoxylin & eosin (H&E), 2) Masson’s Trichrome (TRI), and 3) Immunohistochemistry (IHC). Wound bed biopsies were excised at days 3, 7, 14 and 21 post-wounding. The tissue samples were fixed in 10% buffered formalin, processed and embedded in paraffin to prepare tissue blocks. Then, the tissue blocks were sectioned for 5 µm (H&E, TRI) and 3 µm (IHC). All sections were deparafinized and rehydrated conventionally prior to staining. For H&E and Masson’s trichome, the sections were stained with their respective staining kits. For further investigation, immunohistochemistry was performed to visualize distribution and localization of specific antigen or cellular components responsible for wound healing. In this study, three antibodies were used: Collagen I, Collagen III, and fibroblast. Following deparaffinization and dehydration processes, the sections undergone antigen retrieval process using citrate-based solution in microwave for 10 min and blocking process in 0.3% H_2_O_2_ in methanol for 30 min. Subsequently, the sections were incubated with normal serum for 20 min and were incubated with primary antibody diluted in buffer at 4°C. The sections were then incubated for 1 hour with diluted biotinylated secondary antibody and incubated for 30 min with Vectastain ABC reagent. In between of each incubations, the sections were washed in buffer for 5 min. Following the incubation with Vectastain, the antibody binding sites were visualized by incubation with DAB signal stain kit for 15 min and counterstained with Haematoxylin for 2 min.

All the tissue sections were then subjected to clearing and mounting processes. The slides were observed under microscope (Olympus BX3-25ND6, Japan) and photomicrographs were taken at ×20 magnification for two parts; dermis and hypodermis. The stained tissue sections were scored blindly by histological experts using the modified 0 to three numerical scale as described by Abramov et al. (2007) (Table 2) (Abramov et al., 2007). Any difference in scoring was discussed to a consensus.

**TABLE 2:** Scoring system for Hematoxylin & Eosin, Masson’s Trichome and immunohistochemistry staining. Source: (Abramov et al., 2007).

	Variable	Scoring			
		0	1	2	3
H&E	Reepithelization	None	Partial	Complete but immature or thin	Complete and mature
	Fibroblast proliferation	None	Scant	Moderate	Abundant
	Inflammation cell infiltration	None	Scant	Moderate	Abundant
	Neo- vascularization	None	<5 vessels/HPF	6–10 vessels/HPF	>10 vessels/HPF
	Granulation tissue formation	Immature	Mild maturation	Moderate maturation	Fully matured
Masson’s Trichome	Collagen	None	Scant	Moderate	Abundant
Immunohistochemistry	Collagen I	None	Scant	Moderate	Abundant
	Collagen 111	None	Scant	Moderate	Abundant
	Fibroblast	None	Scant	Moderate	Abundant

### Data analysis

All the quantitative data were analysed using SPSS version 23. Data were presented as Mean ± Standard Error Mean (SEM). Normality test was performed using Kolmogorov-Smirnov test. One-way ANOVA was conducted followed by Post Hoc Tukey’s test to determine statistical significance. *p* values < 0.05 were considered significant. GraphPad Prism software version 9.0 (GraphPad Software, San Diego, California United States) was used to estimate the area under the curve (AUC) with a 95% confidence interval. The AUC was calculated from the wound contraction percentage *versus* time profiles, which was determined by the area normalized to the baseline for the 21-day period (Gagnon and Peterson, 1998).

## Results

### Macroscopic view of wounds

**FIGURE 3:**
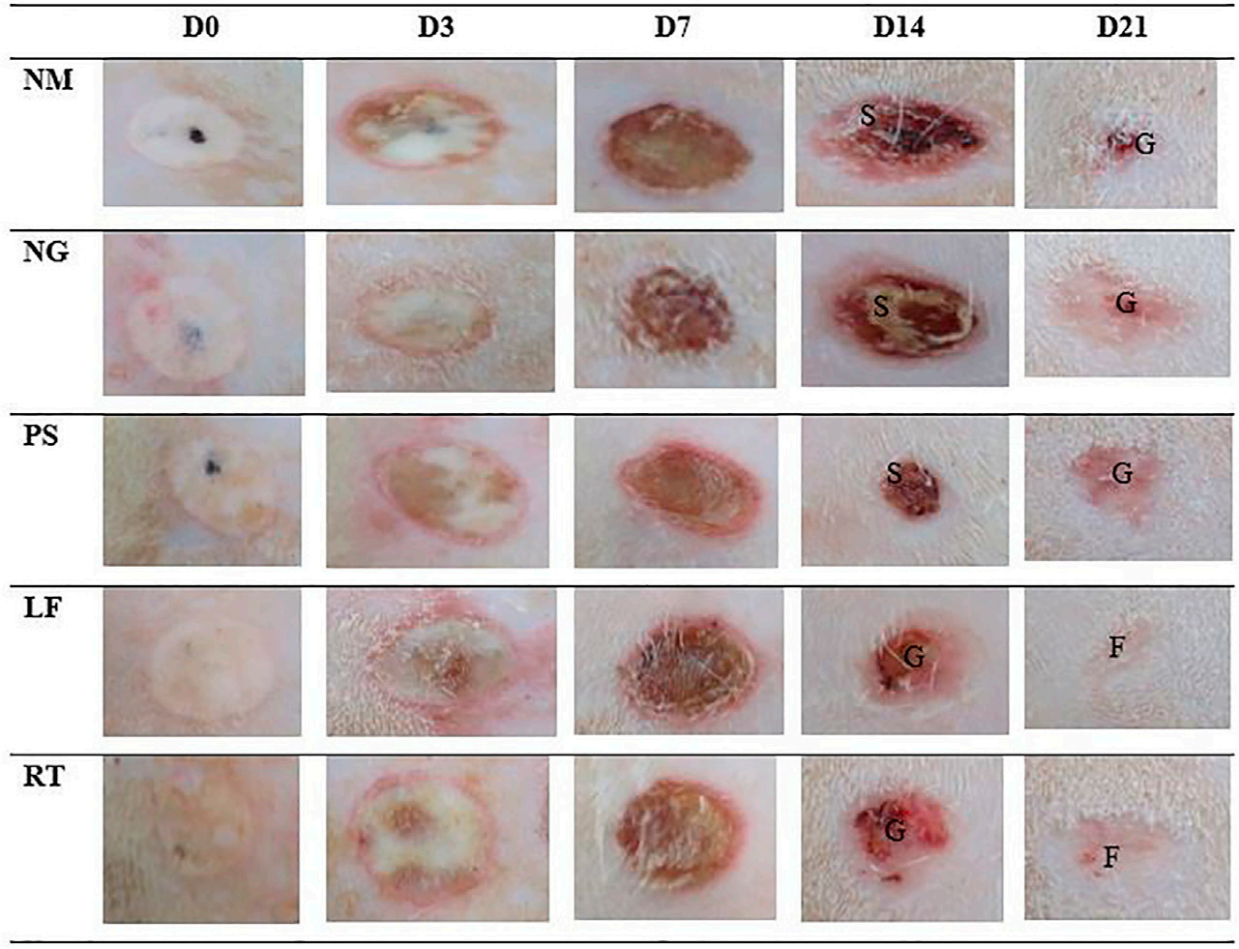
Macroscopic view of five representative male rats from each group at day 0, 3, 7, 14 and 21. S: Scab; G: Granulation tissue; F: Fibrous tissue. NM: normal control group, NG: negative control group, PS: positive control group, LF: LPVa leaf-treated group, RT: LPVa root-treated group.

Macroscopic changes over time for Day 0, 3, 7, 14, and 21 for superficial partial thickness of second-degree burn wounds were shown in Figure 3. In general, immediately after the burn induction, all wounds were non-uniformly round and white in color. Macroscopically, no difference was observed between all the groups following the burn induction, which was reflected by the similar score (score 0) for macroscopic evaluation at day 0 (Table 3). On Day 3, the edemas were more prominent compared to the Day 0, with the score 1 (minimal edema), and the wounds were slightly red with score 1 in all groups. On Day 7, the scabs on the wounds gradually appeared drier and smaller in all the groups, indicated by the score 2 (completely red) in the macroscopic evaluation. While for edema, all groups showed similar score of 2 (clearly distinctive). On Day 14, all control groups demonstrated that the scabs were still present as dark red in color and still attached to the wounds, which represented by the score 2 (completely red) for macroscopic evaluation. Meanwhile, for LF and RT groups, the scabs were mild red in color, or the scabs had dropped off, which represented by the score 0 (none) and 1 (slightly red). On Day 21, the wounds appeared to have almost disappeared in LF and RT groups, with whitish appearance (fibrous tissue) and represented by the score 0 (no redness) in the macroscopic evaluation. Meanwhile, for all control groups, the wounds were still present with pinkish appearance, which indicated granulation tissue and represented by the score 1 (slightly red) for macroscopic examination.

**TABLE 3:** Scoring from macroscopic evaluation at Day 0, 3, 7, 14, and 21. Please refer Table 1 for the scoring system.

Groups	Redness					Edema				
	DO	D3	D7	D14	D21	DO	D3	D7	D14	D21
NM	0	1	2	2	1	0	1	2	2	0
NG	0	1	2	2	1	0	1	2	2	0
PS	0	1	2	2	1	0	1	2	2	0
LF	0	1	2	1	0	0	1	2	0	0
RT	0	1	2	1	0	0	1	2	1	0

### Wound contraction percentage

**FIGURE 4:**
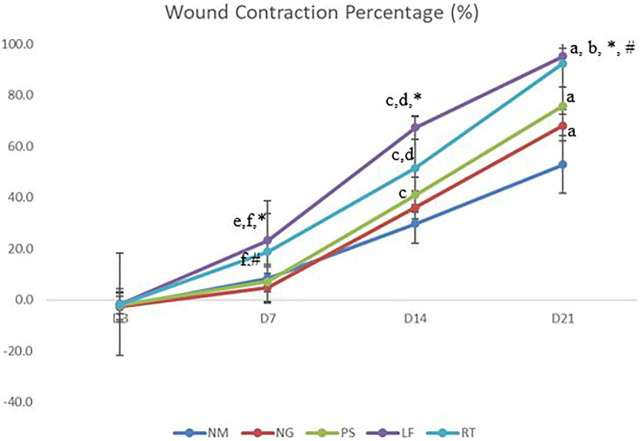
The percentage of wound contraction. NM: normal control group, NG: negative control group, PS: positive control group, LF: LPVa leaf-treated group, RT: LPVa root-treated group. Data were expressed as mean ± SD. Statistically significant results were indicated as **(A)** *p* ≤ 0.05 compared to NM group, **(B)** *p* ≤ 0.05 between NG group, **(C)** *p* ≤ 0.05 compared to NM group, **(D)** *p* ≤ 0.05 compared to NG group, **(E)** *p* ≤ 0.05 compared to NM group, **(F)** *p* ≤ 0.05 compared to NG group, *p ≤ 0.05 compared to LF and PS groups, #*p* ≤ 0.05 compared to RT and PS groups.

On Day 3, all the groups did not show any significant value and demonstrated mildly negative percentage of wound contraction (Figure 4). At Day 7 and 14 of the wound healing, leaf extract group have shown significantly higher percentage of wound contraction when compared to normal, negative and positive control groups. On Day 7, root extract group have shown significantly higher percentage of wound contraction when compared to negative and positive control groups. Meanwhile, on day 14, root extract group have shown significantly higher percentage of wound contraction when compared to negative and normal control groups. Additionally, on Day 14, positive control group had shown significantly higher wound contraction percentage compared to normal control group. For Day 21 of the wound healing, leaf extract and root extract, as well as positive control groups have shown significantly higher wound reduction percentage when compared to normal control group. Most of the wounds were almost healed on Day 21 with leaf and root extract have the highest range of wound percentage (90%–95%).

### Area under the curve

**FIGURE 5:**
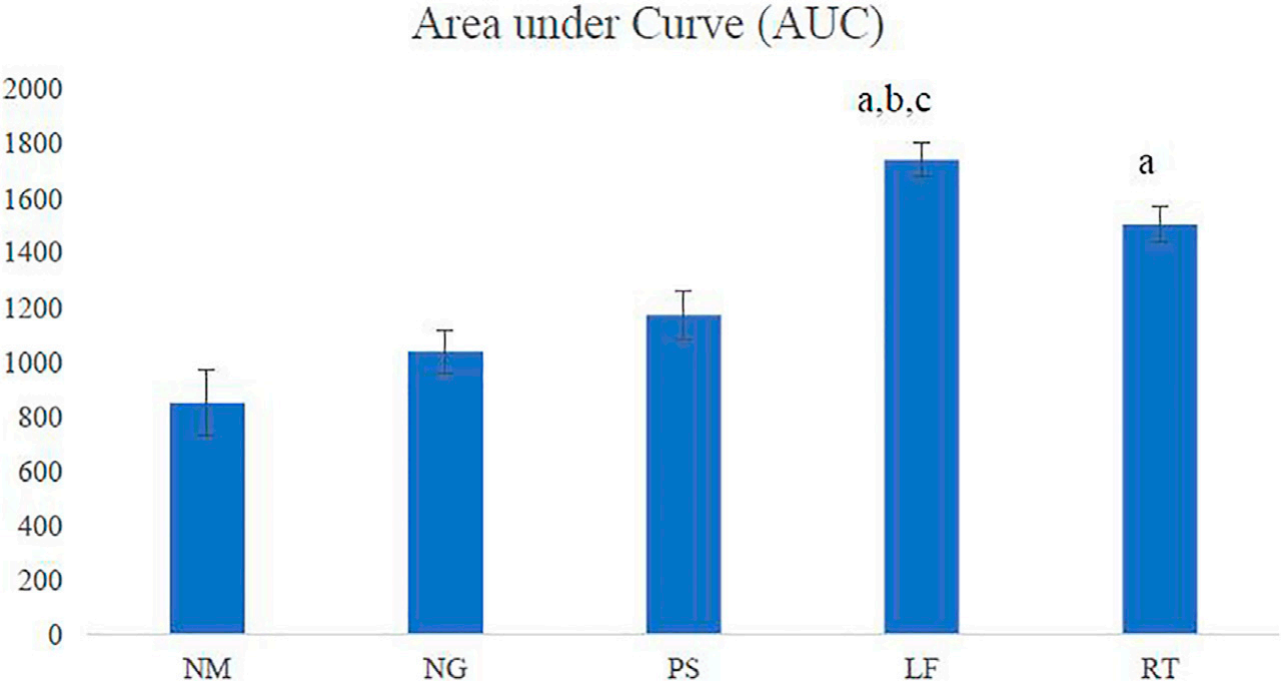
Area under the curve (AUC). NM: normal control group, NG: negative control group, PS: positive control group, LF: LPVa leaf-treated group, RT: LPVa root-treated group. Data were expressed as AUC±SEM. Statistically significant results were indicated as **(A)** *p* ≤ 0.05 compared to NM group, **(B)** *p* ≤ 0.05 between NG group, **(C)** *p* ≤ 0.05 compared to PS group.

The LF group demonstrated the highest AUC among all the groups and showed significant difference when compared to normal (NM), negative (NG) and positive (PS) control groups. The RT group showed significant difference when compared to the NM group (Figure 5).

### Hydroxyproline content

**FIGURE 6:**
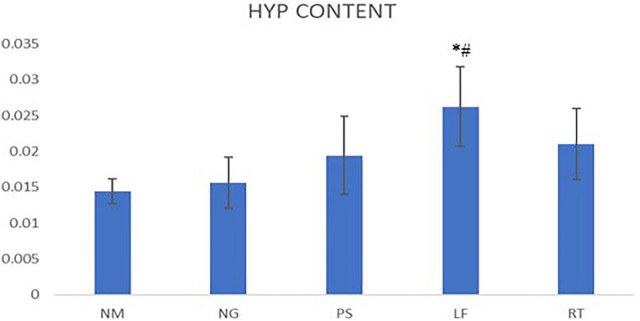
Hydroxyproline contents for wounds Statistically significant difference were indicated as *p ≤ 0.05 between NM group and ^#^ *p* ≤ 0.05 between NG group. NM: normal control group, NG: negative control group, PS: positive control group, LF: LPVa leaf-treated group, RT: LPVa root-treated group.

On day 14, the hydroxyproline content of LF group demonstrated significant difference when compared to NM and NG groups. For the PS and RT groups, the hydroxyproline content were increased but did not reach significant value (Figure 6).

### Histomorphology assessments

**FIGURE 7:**
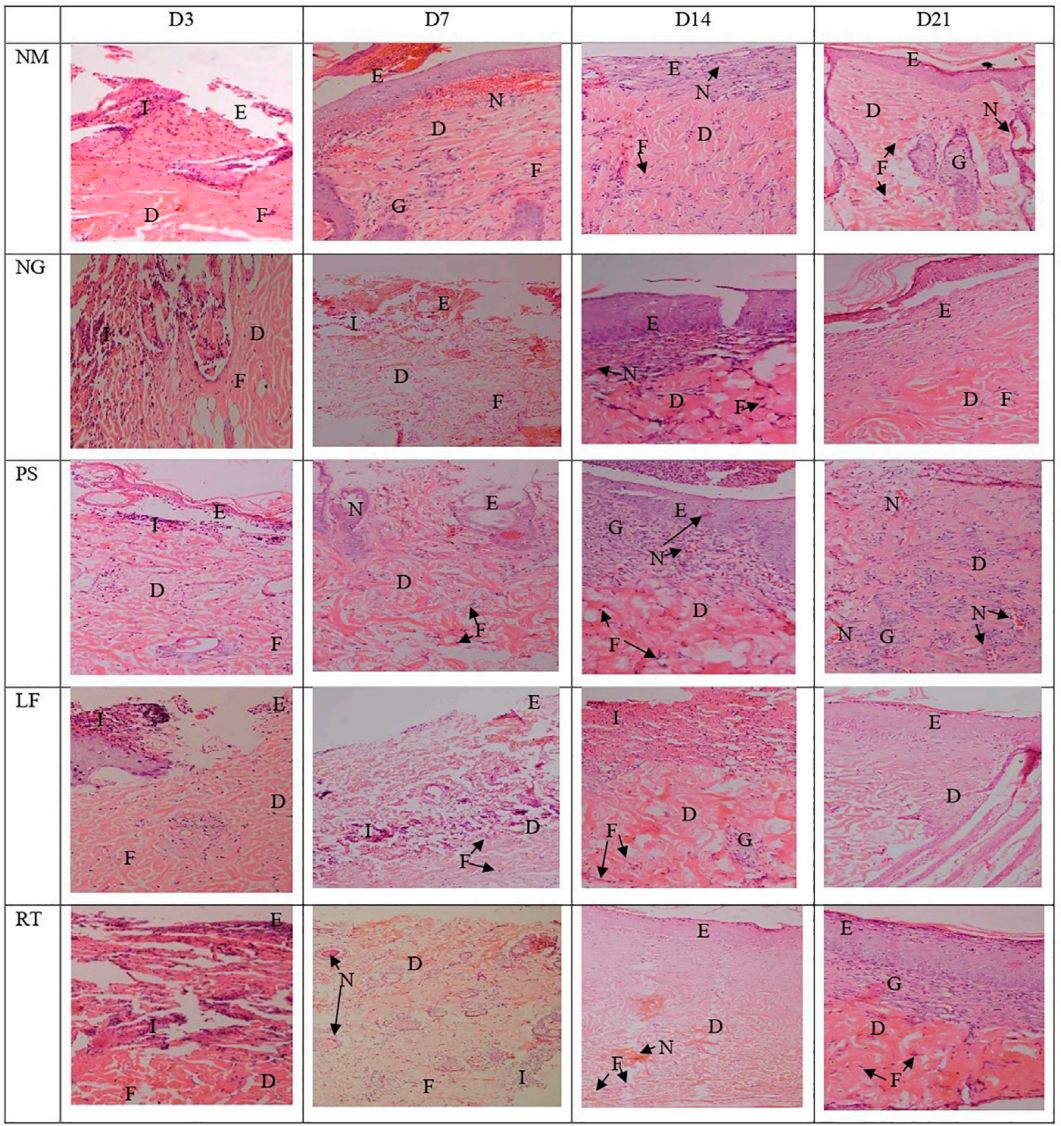
Histopathological view of the burn wounds at Day 3, 7, 14 and 21. Stained with H&E, at ×200 magnification. At day 3, a complete destruction of epidermis layer and partial destruction of dermis layer were observed in all groups, with moderate inflammatory cells and scant fibroblast cells. At Day 21, the re-epithelization in most groups were improved, with abundant fibroblast proliferation, neo-vascularization, and granulation tissue infiltration. E: Epidermis; D: Dermis; I: Inflammatory cells; F: Fibroblast cells, G: Granulation tissue; N: Neovascularization. NM: normal control group, NG: negative control group, PS: positive control group, LF: LPVa leaf-treated group, RT: LPVa root-treated group.

Histopathological view of the burn wounds for all the five groups stained with haematoxylin and Eosin (H&E) were shown in Figure 7. At Day 3, all the groups demonstrated complete destruction of epidermis layer and partial destruction of dermis layer, which indicated superficial partial thickness of second-degree burn. This was also reflected by the zero score for re-epithelization in all groups, except for RT group (Table 4). At Day 21, the re-epithelization in all the groups had improved to score 3, except for RT group which scored 1. All the groups showed scant amount of fibroblast proliferation initially at Day 3, which progressed to abundant amount at Day 21, except for RT group. At Day 7, both the NM and NG groups were still scored as scant (score 1). In terms of inflammation cell infiltration, all the groups demonstrated improvement from Day 3 to Day 21, except for the RT group. Similar patterns were observed for neo-vascularization and granulation tissue formation.

**TABLE 4:** Scoring from histological observations (H&E) of the burn wounds at Day 3, 7, 14 and 21. Please refer Table 2 for the scoring system.

Groups	Reepithelization				Fibroblast proliferation				Inflammation cell infiltration				Neo-vascularization				Granulation tissue formation			
	D3	D7	D14	D21	D3	D7	D14	D21	D3	D7	D14	D21	D3	D7	D14	D21	D3	D7	D14	D21
NM	0	2	2	3	1	2	2	3	1	2	2	3	1	2	2	3	0	2	2	3
NG	0	0	2	3	1	1	2	3	1	1	2	3	0	1	2	3	0	0	2	3
PS	0	0	2	3	1	1	2	3	1	2	2	3	3	1	2	3	0	0	2	3
LF	0	1	2	3	1	2	2	3	2	3	2	3	3	1	2	3	0	1	3	3
RT	0	1	3	3	1	2	2	3	2	2	2	3	1	1	2	3	0	1	3	3

NM: normal control group, NG: negative control group, PS: positive control group, LF: LPVa, leaf-treated group, RT: LPVa, root-treated group.

**FIGURE 8:**
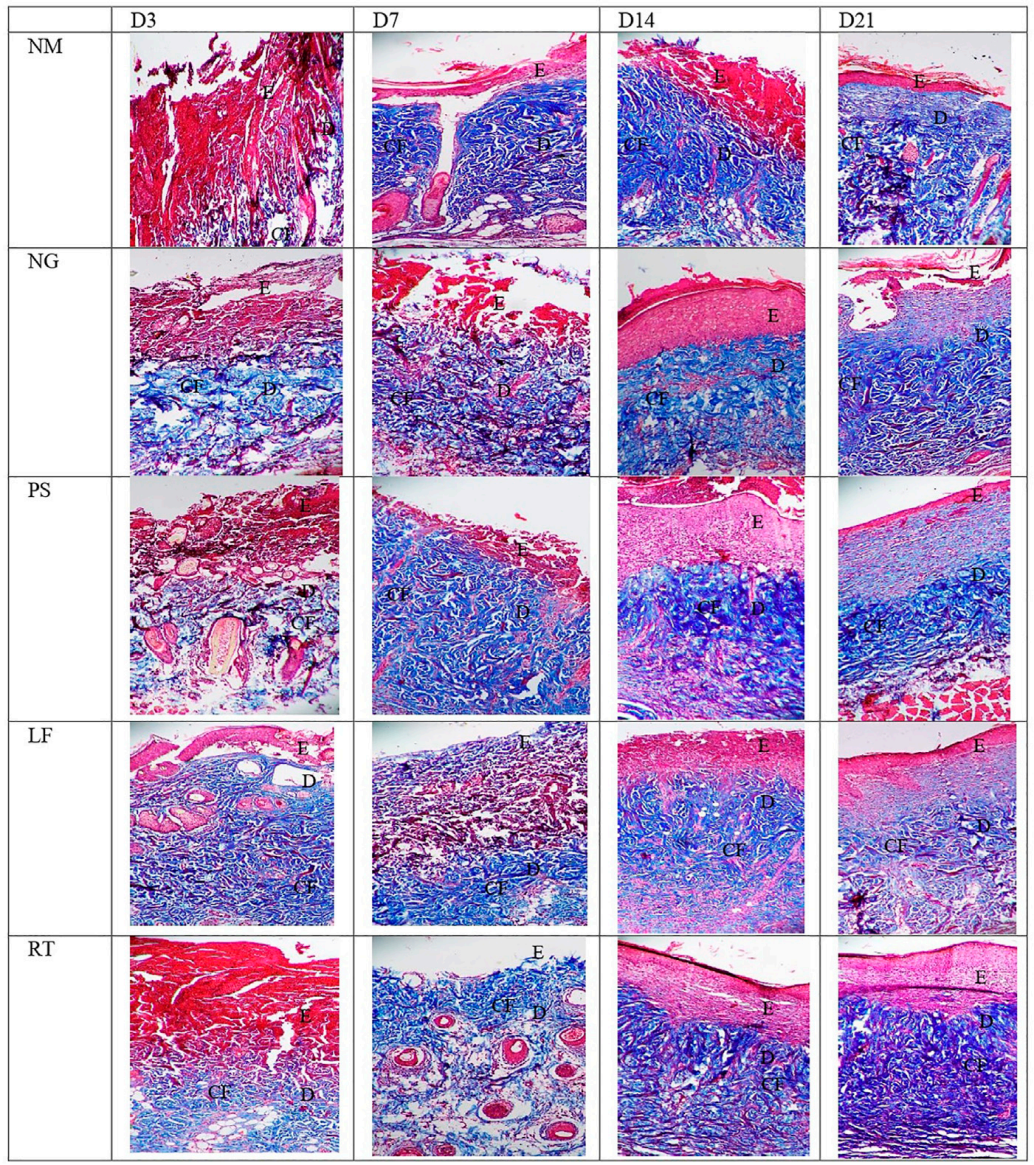
Histopathological view of the burn wounds at Day 3, 7, 14 and 21. Stained with Masson’s trichome staining, at ×200 magnification. Collagen fibers were stained blue, cytoplasm and erythrocyte were stained red, and nuclei were stained bluish brown. Collagen deposition of all groups were abundant, from day 3 to day 21. E: epidermis; D: Dermis; CF: collagen fibers. NM: normal control group, NG: negative control group, PS: positive control group, LF: LPVa leaf-treated group, RT: LPVa root-treated group.

**FIGURE 9:**
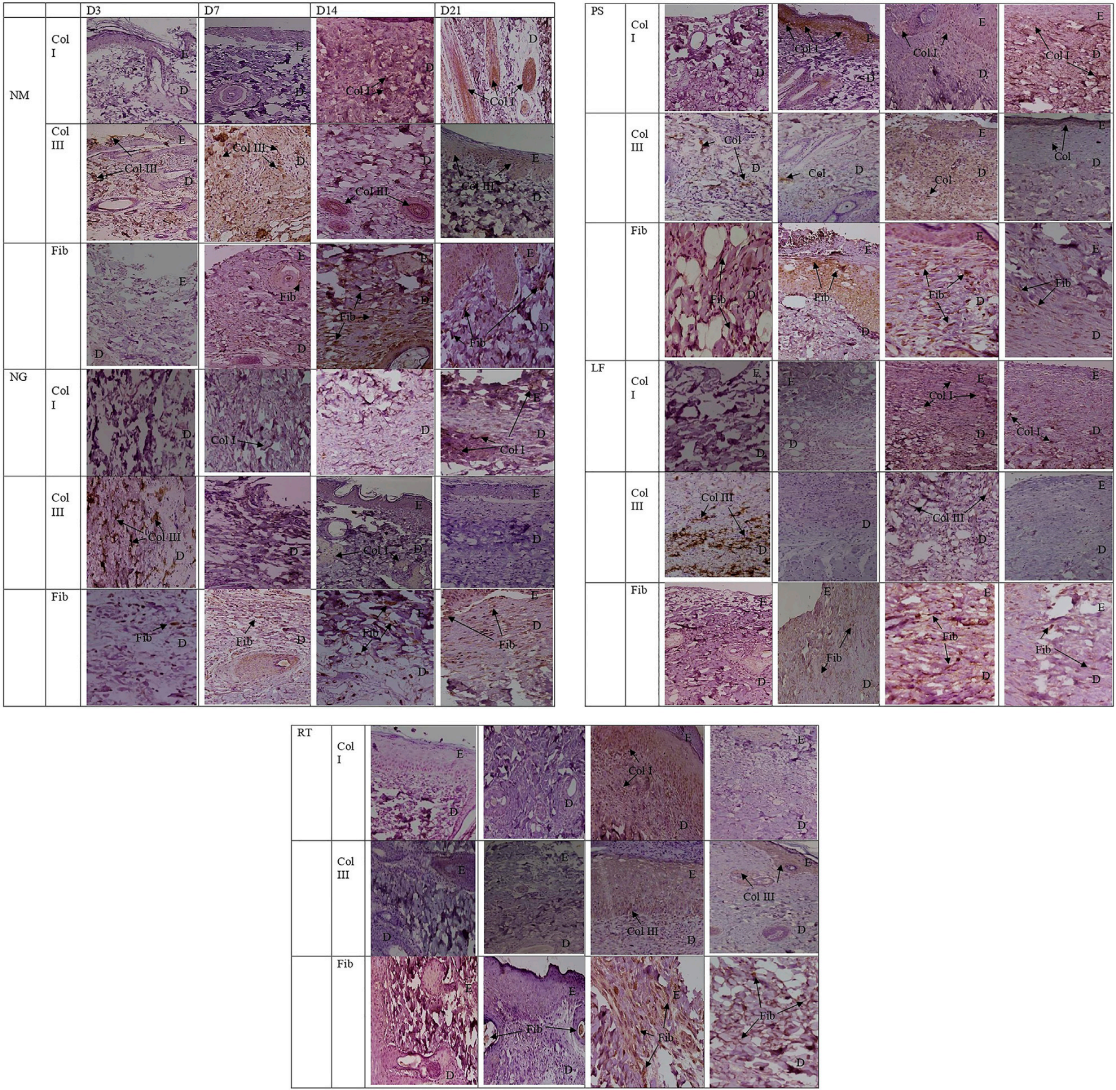
Histopathological view of the burn wounds at Day 3, 7, 14 and 21. Immunohistochemistry staining for collagen I, collagen III and fibroblast, at ×200 magnification. Light brown staining represents positive immunostaining. In most of the groups, collagen III was scant or moderate at day 3, while collagen I were abudant at day 21. Fibroblast were scant at day 3 and were improved to moderate and abundant in most of the groups at day 21. E: epidermis; D: Dermis, Fib: Fibroblast; Col I: Collagen type I, Col III: Collagen type III. NM: normal control group, NG: negative control group, PS: positive control group, LF: LPVa leaf-treated group, RT: LPVa root-treated group.

Histopathological views of the burn wounds for all the five groups stained with Masson’s trichome were shown in Figure 8. Complete destruction of epidermis and partial destruction of dermis were demonstrated at Day 3 post-wounding for all the groups, confirming the success of burn induction in the rat model. In terms of histological scoring, collagen deposition of all the groups for each time points showed similar score of 3, denoting abundant collagen deposition. Therefore, collagen types were differentiated by immunohistochemistry staining (Figure 9). At Day 3, all the groups demonstrated moderate amount of collagen I, except for NM group, which denoted scant amount of collagen I (Table 5). The LF and RT groups showed moderate amount of collagen I from Day 7 to Day 28. Meanwhile, for the other type of collagen, known as collagen III, only the LF group showed moderate amount and RT group scored zero at Day 3. For all the control groups (NM, NG and PS), scant amount of collagen III was noted. However, for RT group, moderate collagen III was noted at Day 14 and Day 21. While, LF group showed scant amount of collagen III since Day 7. Histopathological view of burn wounds using immunohistochemistry staining was also performed for fibroblast (Figure 9). As expected, fibroblasts for all the groups were scant at Day 3 and were subsequently more abundant except for RT root, which paralleled to the H&E staining findings (Table 4).

**TABLE 5:** Scoring for Masson’s trichome staining (collagen deposition) and immunohistochemistry staining (fibroblast, collagen I and III) of the burn wounds at Day 3, 7, 14, and 21.

Groups	Fibroblast				Collagen I				Collagen III				Collagen deposition			
	Immunohistochemistry				Immunohistochemistry				Immunohistochemistry				Masson’s trichom			
	D3	D7	D14	D21	D3	D7	D14	D21	D3	D7	D14	D21	D3	D7	D14	D21
NM	1	2	3	2	1	2	3	3	1	2	1	1	3	3	3	3
NG	1	2	2	2	2	2	2	3	1	1	1	0	3	3	3	3
PS	1	2	3	2	2	2	2	3	1	1	3	2	3	3	3	3
LF	1	2	2	2	2	2	3	2	2	1	1	1	3	3	3	3
RT	1	1	3	3	2	2	2	2	0	0	2	2	3	3	3	3

NM: normal control group, NG: negative control group, PS: positive control group, LF: LPVa, leaf-treated group, RT: LPVa, root-treated group.

## Discussions

Oxidative stress plays an important role in burn wound conversion, in which the zone of stasis cannot be rescued and progresses to necrosis. Reduction in oxidative stress may halt or arrest burn injury progression into deeper tissue (Wardhana and Halim, 2020). Burn wound is also associated with release of mediators such as reactive oxygen species (ROS) and reactive nitrogen species (RNS) which ultimately contribute to local and distant pathophysiological events observed in burn cases. Treatments with antioxidant therapy could be useful to minimize injury in burned patients (Parihar et al., 2008). Xi et al. (2018) reported that LPVa leaves contained various flavonoids such as catechins, rutin, naringin, and myricetin. These compounds have been identified as natural antioxidants that may reduce oxidative stress within the human body. These flavonoids exhibited high 2,2-diphenyl-1-picrylhydrazyl (DPPH) and 2,2′-azino-bis (3-ethylbenzothiazoline-6-sulfonic acid) (ABTS) radical scavenging abilities (Xi et al., 2018). These high radical scavenging abilities may contribute to the antioxidant activity on burn injury and promote formation of scabs and wound contraction, when treated with LPVa leaf extracts. This is in parallel with our findings on wound contraction percentage and the area under the curve, where LPVa leaf extract demonstrated significantly higher value compared to normal control, negative control and positive control rat groups.

The LPVa root extract was as effective as the leaf extract in terms of wound contraction percentage and area under the curve. According to Karimi et al. (2013), the root extract of LPVa contains saponin (Karimi et al., 2013), a bioactive compound that possess beneficial properties related to wound healing including anti-inflammatory, antimicrobial and antioxidant activities (Rao and Gurfinkel, 2000). Similarly, curcumin, a natural polyphenolic molecule extracted from the rhizome of Curcuma longa, also possess anti-inflammatory, antibacterial and antioxidant properties and it demonstrated wound healing properties (Ibrahim and Wong, 2018; Alven and Nqoro, 2020). Curcumin was involved in various stages of the healing process including wound contractions, granulation tissue formation, collagen deposition and tissue remodelling (Zhao et al., 2019). Therefore, the wound contraction ability of LPVa root extract might be due to the saponin content, exerting beneficial effects on wound healing. Karimi et al. (2013) reported that LPVa root also contained kaempferol and myricetin, which are both flavonoids and strong antioxidants (Karimi et al., 2011).

Both leaf and root extracts of LPVa were as effective as silver sulfadiazine, which is the standard burn wound treatment and positive control in this study. As expected, silver sulfadiazine had shown significant wound contraction percentage when compared to normal control at Day 14 and Day 21, but was not significant at early phase of healing (day 7). The silver component is effective in eliminating pathogens *via* direct interactions with bacterial cell membranes, DNA, enzymes and proteins (Zhu et al., 2014). This antimicrobial activity of silver-containing formulation depends on the surrounding environment that drives the release of Ag^+^ ions and other formulations such as sorbents, biologically active compounds and biomolecules (Nešporová et al., 2020). However, silver sulfadiazine failed to produce significant wound contraction percentage from Day 7, as demonstrated by both root and leaf extracts, as well as normal and negative controls. This may have shown that the silver sulfadiazine had delayed wound healing during the early phase of healing process. This could be attributed by a slow progression of silver sulfadiazine in re-epithelization (Aziz et al., 2012), which lead to impaired wound healing at the early stage. Re-epithelialization is an important process to cover damaged epithelial surface as barrier breach offers an entry for wound infection (Pastar et al., 2014). Additionally, repeated usage of silver sulfadiazine has been associated with the formation of pseudoeschar over the affected area which may prevent adequate assessment of the burn wound. In some cases, mechanical debridement is required to remove the pseudoeschar, which is often painful (Lo et al., 2008). Moreover, the insignificant wound contraction percentage of silver sulfadiazine could have been attributed to silver constituent that impair healing during the early phase. The silver constituents have preference to bind to DNA nucleic acid bases and may inhibit replication of the cells during wound healing (Lansdown, 2002a; Lansdown, 2002b). Contradictorily, it was found that LPVa extracts were non-cytotoxic as observed in an *in vitro* cytotoxicity study on L292 rat’s fibroblast cell. Moreover, a repeated dose of LPVa extracts for 28-days in an *in vivo* dermal toxicity study had shown a non-observed adverse effect level (NOAEL) of up to 1,000 mg/kg, indicating that the LPVa extract is safe for skin application with appropriate concentration (Ali, 2014). Additionally, LPVa had also demonstrated protection on human skin keratinocytes from photoaging induced by ultraviolet irradiation (Choi et al., 2010). Therefore, these studies demonstrated that LPVa extracts were not toxic but provide protective effects on skin. Macroscopically on Day 14, burn wounds treated with SSD, LPVa leaf and root extracts showed better healing compared to normal and negative controls. As for the normal and negative controls, the crusts were still attached to the wound on the day 14. After the crust has fallen off, granulation tissue characterized by pinkish coloration could be observed. The granulation tissue will slowly be replaced by fibrous tissue, which appeared whitish on the wound. The negative control group showed that at Day 21, the wounds were still pinkish (indicating granulation tissue) compared to LPVa leaf and root treatment groups that appeared more whitish (indicating fibrous tissue).

Hydroxyproline which is a basic constituent of collagen is a good marker for wound healing assessment. It is one of the most abundant amino acids in collagen and its concentration indicates the concentration of collagen. High concentration of hydroxyproline symbolises faster rate of wound healing (Dwivedi et al., 2016). In this study, positive control, LPVA leaf and root groups have shown increased hydroxyproline content that might reflect increased cellular proliferation and increased collagen synthesis. Among these three groups, the LPVa leaf group showed significantly higher hydroxyproline concentration compared to normal and negative control groups. This result was supported by Chua et al. (2011), which reported that LPVa leaves contain various flavonoids (quercetin, myricetin and kaempferol) and phenolic acids (salicylic acid, vanillic acid, gallic acid, coumaric acid, caffeic acid and chlorogenic acid) (Chua et al., 2011) that could promote collagen synthesis (Chua et al., 2012). Collagen deposition is important for wound strength, cell shape and differentiation (Schultz GS and Moldawer, 2011). Additionally, Karimi et al. (2011) reported that LPVa leaf extract has superior antimicrobial effect (Karimi et al., 2011) which might contribute to the better collagen content. In a previous study by Liang et al. (2019), antibacterial hydrogel dressings applied on mouse full-thickness wound model have improved the granulation tissue thickness and collagen deposition, which also suggests that the antibacterial effect could promote collagen content (Liang et al., 2019).

Histological analyses of all staining used in this study revealed complete destruction of epidermis and partial destruction of dermis for all the groups, observed at Day 3 after wound induction. These characteristics confirmed second-degree or superficial partial thickness of burn wound (Lanham et al., 2020). Involvement of superficial dermis produces the red and wet appearance of wound with blisters. The healing process typically lasted for 3 weeks with minimal scarring (Tolles, 2018), which could be observed by the histological analysis of H&E staining. All the groups received the highest score of three for all the H&E histological scoring at Day 21, indicating that the wounds have completely healed.

As for the scoring of Masson’s trichome staining, all the groups received the highest score of 3, indicating the presence of complete and mature collagen in the wounds of all the groups. Most collagens found in the skin are type I and III, that plays an important role of attracting fibroblast and keratinocytes to the wound (Barbul et al., 2015). The dermis layer of skin could be divided into two layers: papillary and reticular. Papillary is superficial and is structured by flowing connective tissue including reticular, elastic, and non-organized collagen (mostly type III) fibers and capillaries. Meanwhile, reticular is deep and represented by compact connective tissue, which has crosslinked elastic and well-organized collagen fibers (type I and III) and large blood vessels (Arda et al., 2014). In the recent study, the collagen deposition observed in the Masson’s trichome were further differentiated into collagen I and collagen III by immunohistochemistry staining.

Collagen III is the type of collagen synthesized during the early stages of wound healing and is progressively replaced by the dominant skin collagen (collagen I) (Mathew-Steiner et al., 2021). This condition was parallel to our study where at day 21, all three control groups (normal, negative and positive controls) were scored at 3, indicating complete and mature collagen I. However, for both treatment groups (LPVa leaf and root), the scoring was 2, indicating complete but immature or thin collagen I. Both LPVa leaf and root groups have shown complete but immature or thin collagen A except for Day 14 of LPVa leaf group. This might indicate that leaf extract was better at promoting skin collagen synthesis. This was in agreement with the findings by Choi et al. (2010), which reported that LP extract was able to restore skin pro-collagen of human dermal fibroblast cell line that has been destroyed by ultraviolet radiation (Choi et al., 2010).

Fibroblast is the cell responsible for the production of extracellular matrix and collagen, and therefore plays an important role during tissue repair. The migration and proliferation of fibroblast to the wounded area may initiate proliferative phase of repair which subsequently promote effective wound healing and wound contraction. Based on the H & E staining of this study, the LPVa leaf group demonstrated complete and mature fibroblasts at Day 21 compared to the LPVa root group. The superior effect of leaf extract for fibroblast proliferation was in parallel to the hydroxyproline concentration measurements. In fact, fibroblasts in wound healing areas may proliferate and are encircled by collagen fibrils. The new growth-initiating factor for fibroblasts are present and may attach to collagen fibrils at the wound healing site, generating peptides of collagen including prolyl-hydroxyproline (Sato et al., 2020). The prolyl-hydroxyproline which is the main food-derived collagen peptides present in human blood plasma may contribute to chemotactic action on fibroblasts, peripheral blood neutrophils, and monocytes, which are responsible for wound healing and inflammation (Shigemura et al., 2009; Asai et al., 2019). Therefore, the superior effect of leaf extract for fibroblast proliferation may subsequently contribute to the increased amount of hydroxyproline.

## Conclusion

Both leaf and root extracts of LPVa could promote the healing of thermal-burn wounds, with leaf extract being more superior in terms of the hydroxyproline content and histological analysis (Haematoxylin & Eosin and immunohistochemistry). Further studies related with molecular aspects should be performed to determine the mechanism of wound healing by LPVa leaf.

## Data Availability

The original contributions presented in the study are included in the article/Supplementary Material, further inquiries can be directed to the corresponding author.

## Abbreviations

ABTS, 2,29-azino-bis (3-ethylbenzothiazoline-6-sulfonic acid); AUC, area under the curve; Col I, Collagen type I; Col III, Collagen type III; DPPH, 2,2-diphenyl-1-picrylhydrazyl; Fib, Fibroblast; H&E, hematoxylin & eosin; IHC, Immunohistochemistry; LF, Leaf; LP, Labisia pumila; LPVa, Labisia pumila var alata; NG, negative control group; RNS, reactive nitrogen species; ROS, reactive oxygen species; PS, positive control group; RT, Root; SEM, Standard Error Mean; SERMs, selective estrogen receptor modulators; SSD, silver sulfadiazine; TRI, Masson’s Trichrome.
